# Equestrian vaulting as an innovative complementary intervention in eating disorders: A pilot study

**DOI:** 10.1192/j.eurpsy.2021.944

**Published:** 2021-08-13

**Authors:** B. Collacchi, F. Cirulli, M. Borgi, I. Monaci, A. Piccotti, S. Renga, L. Dalla Ragione, M. Ettorre, G. Biccheri, F. Rossetti, S. Cerino

**Affiliations:** 1 Center For Behavioural Sciences And Mental Health, Istituto Superiore di Sanità, er for Behavioural Sciences and Mental Health, Rome, Italy; 2 Department Of Medicine, University of Perugia, Perugia, Italy; 3 Psychology, Family Psychotherapy Academy, Rome, Italy; 4 Umbertide Eating Disorder, USL Umbria 1, Perugia, Italy; 5 Sphere, ECOS - EU, Massa Martana, Italy

**Keywords:** eating disorder, psychiatric rehabilitation, equestrian sport

## Abstract

**Introduction:**

Anorexia is a disorder associated with severe disturbances in eating behaviors and related thoughts and emotions (distorted weight perception, body dissatisfaction). Multidimensional integrative treatment approaches are needed to act both on intrapersonal (e.g. nutritional and psychological) and interpersonal (e.g. behavioral and affective) processes.

**Objectives:**

Aim of this pilot project was to develop a 3-months horse-assisted intervention based on Equestrian Vaulting (EV) and tests its suitability and acceptability in patients with anorexia nervosa. Preliminary observations were carried out to assess the effectiveness of this program on body image, interpersonal relationships and communication and in managing anxiety.

**Methods:**

Seven patients in charge of public service specialized in eating disorder participated in the study. EV activities were performed in an Equestrian Centre included horse grooming, gym exercises and horseback sessions.Clinical and psychological tests (SF 36, IPAQ, EDI3, STAI, SCL90) were administered at baseline and at the end of the program.

**Results:**

Increases in body fat and decreases in lean muscle mass were observed. These were accompained by an improvement in participants’ anxiety and relational skills and in the specific disease related symptoms.

**Conclusions:**

Results indicate the potential of EV to help patients with eating disorder regaining awareness of themselves and their body, a critical element for their future reintegration in the contexts of everyday life and society. Although this is a pilot, the protocol developed represents an initial step to promote the application of EV in persons with eating disorders, informing feasibility in the design of larger controlled studies and suggesting critical variables to be targeted.

